# Plant Virology and Next Generation Sequencing: Experiences with a *Potyvirus*

**DOI:** 10.1371/journal.pone.0104580

**Published:** 2014-08-07

**Authors:** Monica A. Kehoe, Brenda A. Coutts, Bevan J. Buirchell, Roger A. C. Jones

**Affiliations:** 1 School of Plant Biology and Institute of Agriculture, Faculty of Science, University of Western Australia, Crawley, WA, Australia; 2 Crop Protection and Lupin Breeding Branches, Department of Agriculture and Food Western Australia, Perth, WA, Australia; Virginia Tech, United States of America

## Abstract

Next generation sequencing is quickly emerging as the go-to tool for plant virologists when sequencing whole virus genomes, and undertaking plant metagenomic studies for new virus discoveries. This study aims to compare the genomic and biological properties of *Bean yellow mosaic virus* (BYMV) (genus *Potyvirus*), isolates from *Lupinus angustifolius* plants with black pod syndrome (BPS), systemic necrosis or non-necrotic symptoms, and from two other plant species. When one *Clover yellow vein virus* (ClYVV) (genus *Potyvirus*) and 22 BYMV isolates were sequenced on the Illumina HiSeq2000, one new ClYVV and 23 new BYMV sequences were obtained. When the 23 new BYMV genomes were compared with 17 other BYMV genomes available on Genbank, phylogenetic analysis provided strong support for existence of nine phylogenetic groupings. Biological studies involving seven isolates of BYMV and one of ClYVV gave no symptoms or reactions that could be used to distinguish BYMV isolates from *L. angustifolius* plants with black pod syndrome from other isolates. Here, we propose that the current system of nomenclature based on biological properties be replaced by numbered groups (I–IX). This is because use of whole genomes revealed that the previous phylogenetic grouping system based on partial sequences of virus genomes and original isolation hosts was unsustainable. This study also demonstrated that, where next generation sequencing is used to obtain complete plant virus genomes, consideration needs to be given to issues regarding sample preparation, adequate levels of coverage across a genome and methods of assembly. It also provided important lessons that will be helpful to other plant virologists using next generation sequencing in the future.

## Introduction

Next generation sequencing (NGS) technologies are fast becoming a popular method to obtain whole plant virus genomes in a relatively short period of time [1]. Their uptake by plant virologists has been slower than by their counterparts in the medical sciences where the applications are extending much further, rapidly approaching the concept of personalized medicine. Such a situation was impossible before the advent of NGS and its' rapid evolution into an affordable and accessible technology now appearing on laboratory bench-tops throughout the world [2], [3]. Because of the ability to use total RNA extractions for NGS, it is becoming increasingly common to use it to sequence complete genomes of plant viruses and still obtain excellent results [4]–[9]. The challenge now lies not in accessing and using NGS technology, but in analyzing and interpreting the very large datasets suddenly at our disposal [1].

*Bean yellow mosaic virus* (BYMV) (family *Potyviridae*, genus *Potyvirus*) is a single stranded positive sense RNA virus that occurs worldwide. It is a virus with an extensive natural host range that encompasses monocots and dicots, and both domesticated and wild plant species [10], [11]. It is transmitted non-persistently by many different aphid species [12]. BYMV causes serious diseases and losses in many cultivated plant species worldwide. For example, early BYMV infection, which causes serious losses, normally results in systemic necrosis and plant death [13]–[15]. In contrast, late infection with BYMV causes black pod syndrome (BPS) in *Lupinus angustifolius* (narrow-leafed lupin) also resulting in damaging losses [16]. Plants with BPS develop characteristic flat, black pods that have little or no seed [17]. It seems likely that both the BPS and systemic necrosis responses are related to presence of hypersensitivy *Nbm-1* gene and another similar resistance gene [15], [18]–[20].

Wylie *et al.* [21] provided evidence for existence of seven BYMV phylogenetic groupings based on coat protein (CP) sequences and the original hosts of the isolates sequenced: one generalist group with a broad host range including monocots and dicots called the general group, and six other specialist groups each named after the original hosts of the isolates within them (broad bean, canna, lupin, monocot, pea, W). Partial CP sequences from BYMV isolates originally from *L. angustifolius* plants with BPS, systemic necrosis or non-necrotic symptoms placed all of them into the general group [16], [21].

This study aims to compare the genomic and biological properties of BYMV isolates from *L. angustifolius* plants with BPS, systemic necrosis or non-necrotic symptoms, and from two other plant species. NGS was used to sequence 22 BYMV isolates, obtained as part a study conducted in 2011 and from previous studies in south-west Australia [16], [19]. Here, we present the results of genome comparisons with the resulting 23 new BYMV genomes and one *Clover yellow vein virus* (ClYVV) genome with 17 genomes retrieved from Genbank, and biological host range studies with seven BYMV and one ClYVV isolates. We also make recommendations based on the lessons learned from our NGS studies which will be useful to plant virologists employing this approach to obtain whole genomes of other plant viruses.

## Materials and Methods

### Isolates and host plants

Seventeen BYMV isolates were collected from *L. angustifolius* plants with BPS (i.e. systemic necrotic stem streaking with black pods) (11) and systemic necrosis (no black pods) (6), and two from *L. cosentinii* plants with mosaic and leaf deformation as part of a 2011 study in south-western Australia [16]. The remaining three BYMV isolates (FB, LMBNN and LP) were from previous studies [19]. They had been maintained as freeze-dried leaf material obtained from the West Australian Plant Pathogen Culture Collection (FB - WAC10051, LMBNN - WAC10094 and LP - WAC10059). The ClYVV isolate was from the same culture collection (WAC10102).

All plants were maintained at 18–22°C in an insect-proof, air conditioned glasshouse. Plants of *L. angustifolius* cvs Jenabillup (partially resistant to BPS), Mandelup (susceptible to BPS) and germplasm accession P26697 (*Nbm-1* gene absent) were grown in washed river sand. Plants of *Nicotiana benthamiana*, *Trifolium subterraneum* cv. Woogenellup (subterranean clover), *Chenopodium amaranticolor*, *C. quinoa*, *Pisum sativum* cv. Greenfeast (pea) and *Vicia faba* cv. Coles early dwarf (faba bean) were grown in steam-sterilised potting mix. Cultures of virus isolates were maintained by serial mechanical inoculation of infective sap to plants of *N. benthamaniana* or *T. subterraneum*. For inoculations to maintain cultures, or as part of experiments, virus-infected leaves from systemically infected plants were ground in 0.1M phosphate buffer, pH 7.2, and the infective sap mixed with celite before being rubbed onto leaves.

For testing by ELISA, leaf samples were extracted (1 g per 20 ml) in phosphate-buffered saline (10 mM potassium phosphate, 150 mM sodium chloride, pH 7.4, Tween 20 at 5 ml/liter, and polyvinyl pyrrolidone at 20 g/liter) using a mixer mill (Retsch, Germany). Sample extracts were tested for BYMV or ClYVV by double-antibody sandwich ELISA based on a modified protocol described by Clark and Adams [22] and according to manufacturer's recommendations. For generic *Potyvirus* testing, samples were extracted in 0.05 M sodium carbonate buffer, pH 9.6, and tested using the antigen-coated indirect ELISA protocol of Torrance and Pead [23]. The polyclonal antiserum to BYMV was from DSMZ (AS-0717), Germany, to ClYVV from Neogen Phytodiagnostics – formerly Adgen, UK (1171-05) and to generic potyvirus from Agdia, USA (SRA27200). All samples were tested in duplicate wells in microtiter plates. Sap from BYMV or ClYVV infected and healthy *T. subterraneum* leaf samples was included in paired wells to provide positive and negative controls. The substrate was *p*-nitrophenyl phosphate at 1.0 mg/ml in diethanolamine, pH 9.8, at 100 ml/liter. Absorbance values at A_405_ were measured in a microplate reader (Bio-Rad laboratories, USA). Absorbance values of positive samples were always more than three times those of the healthy sap control.

### Sequence data

Twenty two BYMV and one ClYVV sample were sent for NGS on an Illumina HiSeq 2000 (Table 1). For BYMV in total there were 11 samples from *L. angustifolius* plants with BPS, six from *L. angustifolius* plants with systemic necrosis and one from a *L. angustifolius* plant with non-necrotic symptoms. The remaining samples consisted of isolates from other *Lupinus* spp. or were isolates from other hosts representing other phylogenetic groups based on Wylie *et al.* [21], including two samples from *L. cosentinii*, one from *L. pilosus*, and one from *V. faba*. The single ClYVV sample was from *T. repens* (white clover). Total RNA was extracted from each sample using a Spectrum Plant Total RNA kit (Sigma-Aldrich, Australia). Following extraction, total RNA was sent to the Australian Genome Research Facility (AGRF) for library preparation and barcoding (24 samples per lane) before 100 bp paired-end sequencing on an Illumina HiSeq2000. For each sample, reads were first trimmed using CLC Genomics Workbench 6.5 (CLCGW) (CLC bio) with the quality scores limit set to 0.01, maximum number of ambiguities to two and removing any reads with <30 nucleotides (nt). Contigs were assembled using the *de novo* assembly function of CLCGW with automatic word size, automatic bubble size, minimum contig length 500, mismatch cost two, insertion cost three, deletion cost three, length fraction 0.5 and similarity fraction 0.9. Contigs were sorted by length and the longest subjected to a BLAST search [24]. In addition, reads were also imported into Geneious 6.1.6 (Biomatters) and provided with a reference sequence obtained from Genbank (JX173278 for BYMV and NC003536 for ClYVV). Mapping was performed with minimum overlap 10%, minimum overlap identity 80%, allow gaps 10% and fine tuning set to iterate up to 10 times. A consensus between the contig of interest from CLCGW and the consensus from mapping in Geneious was created in Geneious by alignment with Clustal W. Open reading frames (ORFs) were predicted and annotations made using Geneious. Finalized sequences were designated as “complete” based on comparison with the reference sequences used in the mapping process, “nearly complete” if some of the 5′ or 3′ UTR was missing but the coding region was intact, and “partial” if all of the 5′ or 3′ UTR and some of the P1 or CP genes were missing.

**Table 1:** Next generation sequencing data from twenty two *Bean yellow mosaic virus* (BYMV) and one *Clover yellow vein virus* (ClYVV) samples.

Plant/Host ID	Symptoms^a^	No. of reads obtained	No. of reads after trimming	No. of Contigs produced (CLC)	Sample sequence ID	Accession number	Contig length (nt) (CLCGW^c^)	Average coverage (CLCGW)	No. of reads mapped to contig of interest (CLCGW)	Length of consensus (nt) (Geneious)	Average coverage (Geneious)	No. of reads mapped to ref. sequence (Geneious)	Length of Geneious+CLCGW consensus (nt) (Geneious)	Genome completenes
*Lupinus cosentinii*	M	12,684,310	12,402,361	387	MD1	HG970847	9,547	10,173	987,972	9,581	10,562	1,002,513	9,285	partial
*L. angustifolius*	SS, SC	31,131,660	29,497,124	1,851	MD5	HG970848	968–2,576 (5)^b^	11–14 (5)	111–359 (5)	9,541	9	894	9,287	partial^d^
*L. angustifolius*	SS, SC	14,342,828	13,995,123	887	MD6	HG970849	2,625; 1,430	6; 7	103; 202	9,544	7	713	9,285	partial^d^
*L. cosentinii*	M, LD	12,068,236	11,791,675	472	MD7	HG970850	9,563	1,780	173,242	9,636	1,821	175,858	9,530	nearly
*L. angustifolius*	SS, SC	10,841,138	10,582,250	802	SP1	HG970851	9,524	446	43,220	9,544	457	43,877	9,528	nearly
*L. angustifolius*	SS, SC	11,348,684	11,076,092	1,098	GB17A	HG970852	9,655	1,723	169,883	9,983	12,313	954,307	9,530	complete
*L. angustifolius*	SS, BP	30,708,142	29,877,487	2,498	GB42C	HG970853	701–1,557 (8)	6–14 (8)	49–191 (8)	9,544	10	1,045	9,390	nearly^d^
*L. angustifolius*	SS, BP	11,780,826	11,532,853	360	GB32A	HG970854	9,593	5,241	511,194	9,915	5,317	511,902	9,538	complete
*L. angustifolius*	VC	11,166,630	10,924,778	476	LMBNN	HG970855	9,533	2,869	278,099	9,544	2,925	279,855	9,531	nearly
*L. angustifolius*	BP, NVSS	14,710,190	14,415,378	820	ES69C	HG970856	554–2,360 (9)	3–11 (9)	19–211 (9)	9,544	8	850	9,369	partial^d^
*L. angustifolius*	MSS, BP	14,308,420	13,991,683	907	ES67C	HG970857	534–1,413 (9)	3–8 (9)	18–101 (9)	9,544	6	660	9,479	nearly^d^
*L. angustifolius*	MSS, BP	15,468,144	15,091,935	837	ES55C	HG970858	9,587	1,631	159,066	9,567	1,656	159,704	9,514	complete
*L. angustifolius*	SS, SC	13,676,576	13,370,007	798	ES11A	HG970859	709–1,417 (9)	767–2207 (9)	8,193–31,962 (9)	10,324	2,708	280,928	9,530	complete
*L. angustifolius*	SS, BP	14,890,148	14,565,374	855	PN83A	HG970860	9,556	7,239	704,254	10,262	7,382	715,978	9,530	complete
*L. angustifolius*	SS, BP	15,789,358	15,445,952	795	PN80A	HG970861	9,532	4,825	468,607	9,625	4,934	475,461	9,530	complete
*L. angustifolius*	SS, BP	16,826,134	16,427,970	919	PN77C	HG970862	646–926 (5)	4–5 (5)	27–50 (5)	9,544	4	471	9,274	partial^d^
*L. angustifolius*	SS, BP	14,713,078	14,380,894	1,643	AR87C	HG970863	9,561	2,600	252,909	9,912	2,587	256,835	9,530	complete
*L. angustifolius*	SS, BP	12,537,974	12,252,296	827	AR98C	HG970864	676–1,264 (5)	6–7 (5)	56–88 (5)	9,544	6	617	9,447	nearly^d^
*L. angustifolius*	SS, BP	16,337,836	15,965,779	1,023	AR93C	HG970865	9,547	5,856	568,049	9,990	6,004	576,414	9,530	complete
*L. pilosus*	VC, SM, LD, Y	17,060,028	16,402,537	889	LP	HG970866	9,521	1,159	113,369	9,034	737	6,866	9,520	nearly
*Vicia faba*	VC, SM, LD	16,293,018	15728187	519	FB	HG970867	9,464	1,035	100,666	-	-	-	9,417^e^	nearly
					LPexFB	HG970868	9,450	603	58,411	-	-	-	9,417^e^	nearly
*L. angustifolius*	SS, SC	15,974,728	15,402,955	720	NG1	HG970869	9,548	4,192	409,456	9,595	4,399	422,536	9,530	complete
*Trifolium repens*	Not recorded	11,385,986	11,121,357	149	ClYVV	HG970870	9,439	6,195	6,195	9,585	65	6,295	9,439	nearly

a Coded symptom description: BP, black pods; LD, distortion; M, mosaic; MSS, mild necrotic stem streaking; NVSS, no visible stem streaking; SS, necrotic stem streaking; SM, severe mosaic; SC, shepherds crook appearance (i.e. bending over and apical tip necrosis); VC, vein clearing; Y, yellowing.

b Numbers in parenthesis represent the total number of contigs for the sample with lengths indicated by the preceding range.

c CLC genomics workbench.

d Indicates that the genome is draft only, meaning less than or equal to ten times average coverage.

e Indicates that the final sequence is derived entirely from CLCGW de novo assembly.

### Phylogenetic analysis

The new sequences were aligned with the 17 retrieved from Genbank using Clustal W in MEGA 5.2.1, prior to phylogenetic analysis [25]. Phylogenetic analysis compared (i) coding regions of all BYMV genome sequences and (ii) coding regions of all BYMV genome sequences except seven with average coverage of 10 times or less. Neighbor-joining trees were made using the number of differences model with a bootstrap value of 1000, Maximum Likelihood trees using the Tamura-Nei model with a bootstrap value of 1000, and Minimum Evolution trees using the number of differences model with a bootstrap value of 1000. Tables of nucleotide (nt) percentage differences were calculated for the complete genomes using the pairwise comparison function with the number of differences model. Final sequences were submitted to the European Nucleotide Archive (ENA) with accession numbers HG970847–HG970870 (Table 1).

### Biological data

For host range studies, seven isolates of BYMV and one of ClYVV were mechanically inoculated onto leaves of *L. angustifolius*, *N. benthamaniana*, *T. subterraneum*, *C. amaranticolor*, *C. quinoa*, *P. sativum* and *V. faba* plants (5 plants/isolate). For each experimental host, uninoculated and mock-inoculated controls were included at time of inoculation (five plants each). There were five isolates from *L. angustifolius*, one from a plant with BPS (AR93C), three from plants with systemic necrosis (MD5, GB17A and ES11A), and one from a plant with non-necrotic symptoms (LMBNN). The remaining isolates were from plants of *L. cosentinii* (MD7) and *L. pilosus* (LP) with non-necrotic symptoms. Symptoms were recorded and samples from inoculated and tip leaves tested by ELISA weekly beginning 7 days after inoculation for up to six weeks.

## Results

### Sequence data

From the single ClYVV and 22 BYMV samples, the numbers of raw reads obtained from NGS were 10,841,138–31,131,660, but these numbers were reduced to 10,582,250–29,877,478 after trimming (Table 1). Following *de novo* assembly of each individual sample using CLCGW, the numbers of contigs produced were 149–2498. Contig of interest lengths were 534–9,655 nt with average coverage 3–10,173 times and the numbers of reads mapped to each contig were 18–987,972. After mapping to a reference genome in Geneious, the lengths of the consensus sequences were 9,034–10,324 nt, with average coverage of 4–12,313 times and the numbers of reads mapped to the references sequence were 471–1,002,513. Final sequence lengths consisted of the consensus of the contig from CLCGW and the consensus from Geneious, and were 9,274–9,530 nt. All samples yielded one sequence of interest, with the exception of FB, which contained a second BYMV sequence which we called “LPexFB”. In all cases, except for ClYVV, the contigs of interest were most closely related to BYMV after being subjected to Blastn analysis. ClYVV was most closely related to the only other ClYVV complete genome available on Genbank. In total, there were nine complete genomes, ten nearly complete genomes (including ClYVV) and five partial genomes.

### Phylogenetic analysis

**Figure 1:**
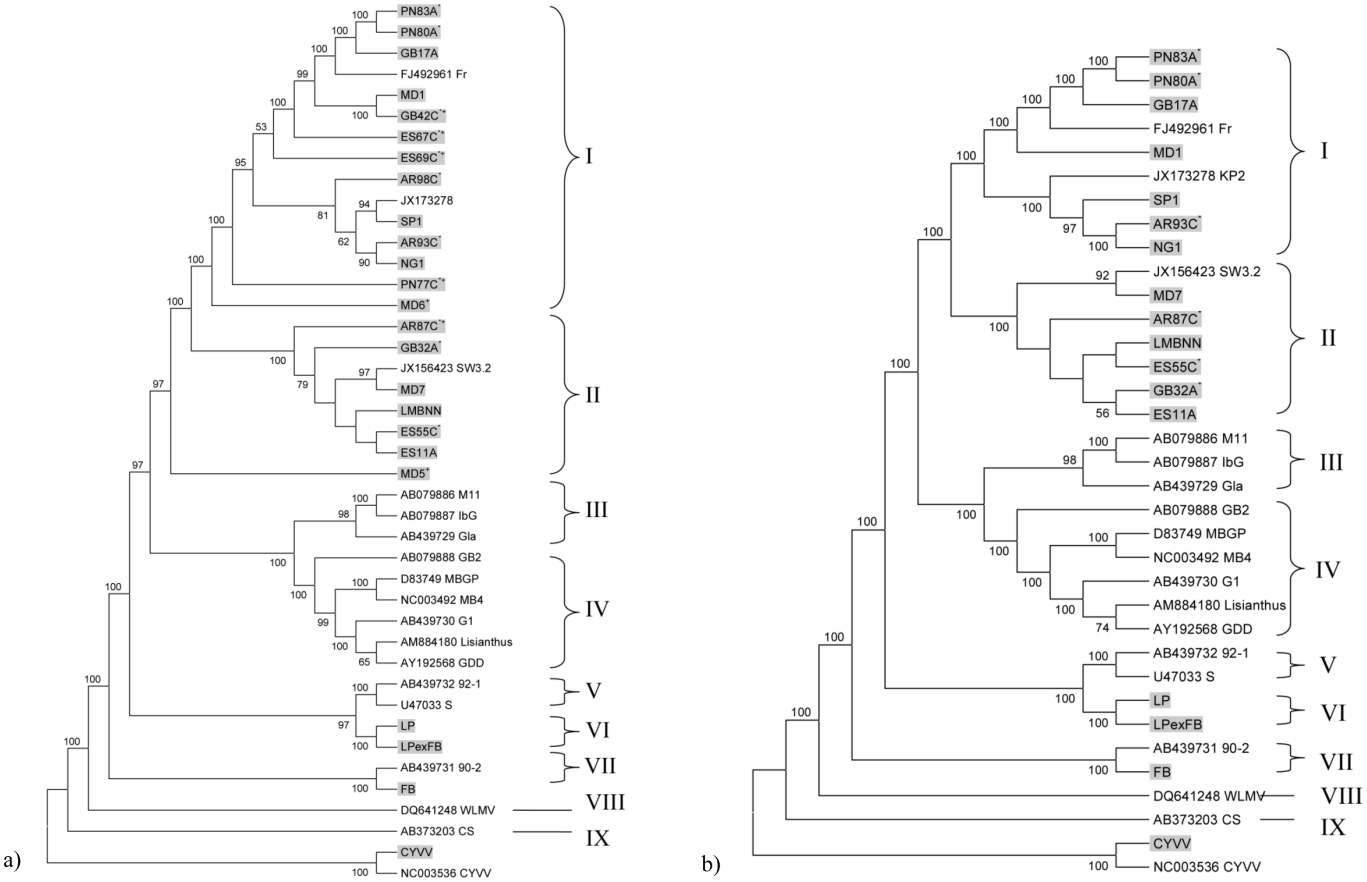
Neighbor-joining relationship phylograms obtained from alignment of the coding regions of *Bean yellow mosaic virus* (BYMV) genomes. The alignments were generated in MEGA 5.2.1 using ClustalW and tree branches were bootstrapped with 1000 replications. The trees were rooted with a sequence of *Clover yellow vein virus* (ClYVV), the closest relative to BYMV. New isolates from this study shown in grey, isolates obtained from *Lupinus angustifolius* plants with BPS are denoted by *, and isolates with genomes designated as “draft” are denoted by +. a) Complete coding regions of BYMV genomes, including draft sequences, with isolates retrieved from Genbank. b) The same sequences as in a) but with draft sequences removed from the analysis.

Phylogenetic analysis comparing the coding regions of 23 new complete or nearly complete BYMV genomes and one new nearly complete ClYVV genome with those of 17 BYMV and one ClYVV genome retrieved from Genbank provided 100% bootstrap support for eight of nine phylogenetic groups (I, II, IV–IX). The remaining group (III) had 98% bootstrap support. Seven of the new genomes had average coverages of less than or equal to ten times (MD5, MD6, GB42C, ES69C, ES67C, PN77C and AR98C) and five of these (MD5, MD6, ES67C, ES69C and PN77C) did not sit well within groups I and II. Although they appear to belong to them, genomes such as MD6 and PN77C sit out on their own, almost separate from the other sequences, leaving groups I and II poorly resolved (Figure 1a). In contrast, when sequences of the seven genomes with poor average coverage (≤10 times) were removed, phylogenetic analysis gave the same results but with much greater resolution between groups I and II and improved bootstrap support for groups I–IX (Figure 1b). Those removed were designated as “draft” genomes because all had low coverage and/or small gaps. When all the genomes, including those with poor coverage were analyzed using Maximum Likelihood or Minimum Evolution methods, the tree topologies shown were the same as the Neighbor-Joining method.

The range of original isolation hosts within each grouping varied (Table 2). Group I consisted of nine sequences from two dicot, and two monocot species. Group II consisted of seven sequences from two dicot and one monocot species. Group III consisted entirely of three sequences from one monocot species. Group IV was made up of three sequences from an unknown original host or hosts, as well as two from a monocot and one from a dicot species. Groups V–IX consisted entirely of dicot species belonging to a single family, and were represented by up to three sequences. All dicot species were from families *Fabaceae* or *Gentianaceae*, and all monocot species were from families *Orchidaceae* or *Iridaceae*.

**Table 2:** Original hosts of isolates within each phylogenetic grouping.

Phylogenetic group (old name)	Accession numbers	Dicot	Monocot
I (general)	FJ492961, JX173278, HG970847, HG970851, HG970851-52, HG970856-57, HG970860-62, HG970864-65, HG970865	*Lupinus angustifolius* a (6)^b^, *L. cosentinii* (1)	*Diuris magnifica* (1), *Freesia* sp. (1)
II (general)	JX156423, HG970848, HG970850, HG970854-55, HG970858-59, HG970863	*L. angustifolius* (5), *L. cosentinii* (1)	*Diuris* sp. (1)
III (monocot)	AB079886, AB079887, AB439729	-	*Gladiolus hybrid* (3)
IV (general)	AB079888^c^, D83749^c^, NC003492^c^, AB439730, AM884180, AY192568	*Eustoma russellianum* (1),	*Gladiolus* sp. (1), *Gladiolus hybrid* (1)
V(faba bean)	AB439732, U47033	*Trifolium pratense* (1), *Vicia faba* (1)	-
VI (lupin)	HG970866, HG970868	*L. pilosus*(1), *Vicia faba* (1)	-
VII (faba bean)	AB439731, HG970867	*V. faba* (2)	-
VIII (W)	DQ641248	*L. albus* (1)	-
IX (pea)	AB373203	*Pisum sativum* (1)	-

a Species from *Lupinus*, *Vicia* and *Trifolium* are from family *Fabaceae*. *Eustoma* is from family *Gentianaceae*, *Gladiolus* and *Freesia* are from family *Iridaceae*, *Diuris* is from family *Orchidaceae*.

b Numbers in parentheses represent the numbers of genomes with from this original isolation host.

c Denotes an unknown original host for that accession number.

### Sequence analysis

When the coding regions of the 16 new BYMV genomes (draft genomes excluded) and one ClYVV genome were analyzed against those retrieved from Genbank, the nt percentage identities within each phylogenetic group were ≥96.6% (I), ≥98.6% (II), ≥93.9% (III), ≥94% (IV), ≥90.7% (V), ≥99.8% (VI), ≥97.6% (VII) and ≥97.5% for ClYVV (Table S1). When the six sequences from *L. angustifolius* plants with BPS were compared to each other their percentage nt identites were ≥93.8%. When the sequences from all *L. angustifolius* plants were compared to each other their percentage nt identities were also ≥93.8%. Across all 33 BYMV sequences used in this analysis the nt identities were ≥75.6%. When the ClYVV sequences were compared to the BYMV sequences, overall the percentage nt identities were 66.4–67.9%.

### Biological data

All seven BYMV isolates and one ClYVV isolate inoculated to plants caused systemic symptoms of varying severity in *N. benthamiana*, *T. subterraneum* and *V. faba* (Table 3). However, apart from ClYVV and BYMV isolate GB17A in *V. faba*, none of them induced systemic necrotic symptoms, which were severe only with ClYVV. In *C. amaranticolor*, ClYVV and five BYMV isolates caused obvious systemic symptoms, while infection was restricted to inoculated leaves with the isolate originally from *L. angustifolius* plants with BPS and another originally from an *L. angustifolius* plant with non-necrotic symptoms. In *C. quinoa*, although all isolates infected inoculated leaves, only ClYVV caused systemic invasion. In contrast, in *P. sativum*, only BYMV isolate LP caused any infection.

**Table 3:** Responses of seven plant species to inoculation with eight different isolates of *Bean yellow mosaic virus* (BYMV) and one of *Clover yellow vein virus* (ClYVV).

Isolate^a^	Original host	Symptoms in original host	*Chenopodium amaranticolor*	*C. quinoa*	*Lupinus angustifolius* cv. Jenabillup	*L. angustifolius* cv. Mandelup	*L. angustifolius* line P26697	*Nicotiana benthamaniana*	*Pisum sativum* cv. Greenfeast	*Trifolium subterraneum* cv. Woogenellup	*Vicia faba* cv. Coles Early Dwarf	Phylogenetic grouping
ClYVV	*T. repens*	Not recorded	LNS^b^, SCS, SVC	LNS, SCS, SVC	SS, N, St, Y, LD	SS, SN, DR	SSS, R, B, M	SM	NI	VC, SM, LD, St	SS, SM, LD, SN	n/a
LMBNN	*L. angustifolius*	VC	LCS	LMCS	SM, DR, B	B, M	B, M	MM	NI	MM	SM, LD	II
AR93C	*L. angustifolius*	SS, BP	LCS	LNS	MSS, SN, St, Y	SSS, DR, SN	NI^c^	M	NI	VC, M, LD, St	MM	I
MD5	*L. angustifolius*	SS, SC	LNS, SCS, SVC	LNS, LCS	SSS, SN	SSS, DR, SN	SM	MM	NI	VC, M, LD	M	II
GB17A	*L. angustifolius*	SS, SC	LCS, SCS	LCS, LNS	M, DR, Y, SSS, N	nt	nt	MM	NI	MVC, SM, LD	SS, M	I
ES11A	*L. angustifolius*	SS, SC	LCS, SCS, SVC	LVC, LCS, NSH	M, DR, Y	B, M	B, M	MM	NI	MVC, MM	SM	II
MD7	*L. cosentinii*	M, DR	LCS, SCS, SVC	LCS	SM, DR, B	PSN, M, B	NI^c^	MM	NI	MVC, M	SM	II
LP	*L. pilosus*	VC, SM, LD, Y	SCS, SVC	LNS	SSS, DR, SN	NI^d^	SSS, SN	M	MM	VC, M, LD, St	M	VI
Uninoculated	n/a	n/a	NI	NI	NI	NI	NI	NI	NI	NI	NI	n/a
Mock	n/a	n/a	NI	NI	NI	NI	NI	NI	NI	NI	NI	n/a

Leaves were inoculated with infective sap. Samples from inoculated and tip leaves were tested by ELISA for BYMV, ClYVV and potyviruses in general.

a Locations where isolates were collected from: ClYVV, New South Wales (NSW); LMBNN, Mt. Barker, Western Australia (WA); AR93C, Arthur River, WA; MD5 and MD7, Medina, WA; GB17A, Gibson, WA; ES11A, Esperance, WA; LP, South Perth WA.

b Coded symptom descriptions: B, bunchy new growth; BP, black pods; DR, leaf drop; LCS, local chlorotic spots; LD, leaf distortion; LMCS, local mild chlorotic spots; LNS, local necrotic spots; M, mosaic; MM, mild mosaic; MSS, mild necrotic stem streaking; MVC, mild local vein clearing; NI, not infected; NSH, local necrotic spots with halo; nt, not tested; PSN, partial necrotic stem streaking (no infection in uninoculated leaves); R, reddening; SC, shepherds crook appearance (i.e. bending over and apical tip necrosis); SS, necrotic stem streaking; SSS, severe necrotic stem streaking leading to plant death; SCS, systemic chlorotic spots; SM, severe mosaic; SN, severe necrosis of uninoculated leaves and new growth; St, stunting; SVC, systemic vein clearing; VC, localized vein clearing; Y, yellowing.

c Denotes that the initial round of inoculations failed to infect plants of this species, and due to a lack of seed the inoculations were not repeated.

d Denotes that two rounds of inoculation failed to infect *L. angustifolius* cv. Mandelup. Further testing involving sap, aphid and graft inoculations are required.

In *L. angustifolius* cvs Jenabillup and Mandelup, three BYMV isolates caused systemic non-necrotic symptoms. These were originally from plants of this species with non-necrotic symptoms (LMBNN) or systemic necrotic symptoms (ES11A), and *L. cosentinii* (MD7) from a plant with mosaic and leaf distortion. All other BYMV isolates and the ClYVV caused systemic necrotic symptoms in cvs Jenabillup and Mandelup. In accession P26697, with ClYVV and four BYMV isolates for which symptom data are available, the reactions resembled those in cv. Jenabillup, with the exception of MD5 which produced severe mosaic (i.e. non-necrotic) symptoms instead of systemic necrosis. Isolates LBMNN and ES11A caused non-necrotic symptoms, while ClYVV and LP caused systemic necrosis. Failure of isolates AR93C and MD7 to infect P26697 probably represents escapes, but there was no seed left of P26697 for further testing. Isolate LP did not infect *L. angustifolius* cv. Mandelup on two separate occasions by sap inoculation, but further inoculations using grafting or aphids would be needed to establish if this is a resistance reaction.

## Discussion

Before this study was conducted, there were only 17 complete BYMV genomes on Genbank. The ten complete and eight nearly complete genomes from this study doubled available BYMV genomic data in the database. Moreover, the five additional partial genomes we obtained will be useful in future studies. Our genome results enabled the phylogenetic makeup of BYMV to be examined thoroughly, revealing presence of nine distinct groups, including the subdivision of the former generalist group into three new groups. We recommend replacing the phylogenetic groupings of Wylie *et al.* [21] with numbered group names (I–IX). We have not included one former specialist group based on CP genes, the canna group, in our analysis because it was not represented by any whole genome sequence. Use of whole genomes revealed that the previous phylogenetic grouping system based on partial genome sequences and original isolation hosts was unsustainable. This is because genome sequences from broad bean are present in two former specialist groups (now V and VII), from various *Lupinus* species in two former specialist groups (now VI and VIII), and two former generalist groups (now I and II). Moreover, although we have not re-analyzed sequences of CP genes, Wylie *et al.* [21] had previously placed a CP sequence from the dicot species *Eustoma russellianum* (family *Gentianaceae*), in the former monocot group (now III). Numbering of groups prevents such confusion arising from use of natural isolation host names. Our results highlight the importance of using complete genomes wherever possible to define phylogenetic groupings. The results also highlight the need for further sequencing and analysis of BYMV isolates likely to belong to former specialist phylogenetic groupings, which will provide greater insight into the genetic makeup of BYMV.

Close examination of the nt percentage sequence identities between BYMV and ClYVV genomes revealed that the divergence between them is greater than previously thought. Overall, BYMV percentage nt identities ranged from 75.6 to 99.5%. The species demarcation for potyviruses is currently 23–24% divergence at the nt level [26], and some of the BYMV isolates compared came close to this. The two ClYVV genomes shared 97.5% nt identity, but when compared to all the BYMV genomes, nt identities were 66.4–67.9%, well beyond the species demarcation point for potyviruses. ClYVV was originally considered an isolate of BYMV but was later shown to be a distinct virus [26], [27], [28]. Our percentage identities support that distinction. However, some BYMV phylogenetic groups were more closely related to ClYVV than others. For example when compared with all other BYMV sequences, the single sequences from groups VIII and IX had percentage identities of just 78.4–79.8% and 75.6–76.9% to BYMV respectively, whereas when compared to ClYVV their nt percentage identities were 67.0–67.7% (Table S1). Again, further genome sequences from these groups and ClYVV are required for a more conclusive analysis.

Based on our phylogenetic and sequence analyses, BYMV isolates associated with BPS in *L. angustifolius* were not different phylogenetically from other BYMV isolates we sequenced from *L. angustifolius*, *L. cosentinii*, or other hosts within the same phylogenetic groups (I and II). Also, from the host data from our inoculations, there was no host reaction that could be used to distinguish a particular isolate as causing BPS. However, there were some other interesting differences. Although isolate ES11A behaved in a similar manner to isolate LMBNN, which overcomes the *Nbm-1* hypersensitivity gene in *L. angustifolius* plants [19], [20], it was isolated from a *L. angustifolius* plant originally displaying systemic necrosis. ClYVV behaved like isolate LP, but whether ClYVV interacts with both *Nbm-1* and the second putative BYMV hypersensitivity genes, or unknown ClYVV-specific genes in *L. angustifolius*, is not clear [19]. ClYVV and all group I and II isolates failed to infect *P. sativum* cv. Greenfeast although the group VI isolate LP did cause infection. This may be due to the fact that this cultivar, like many commercial pea cultivars, may contain the BYMV resistance gene *mo* and ClYVV resistance genes *cyv* or *cyv-2* [29], [30] and their responses are strain specific. Induction of severe necrotic symptoms in *V. faba* by ClYVV but not the BYMV isolates is expected, as this is the classical method for distinguishing BYMV from ClYVV [10], [20].

In this study, we used NGS to obtain complete virus genomes and it proved both an advantage and a disadvantage over traditional sequencing methods. It allowed large amounts of data to be generated quickly, but analysis of the data proved a major challenge. Many free programs exist for the assembly of NGS data (e.g. Velvet, SOAP de novo, Abyss and bowtie) but they all require the researcher to be proficient in the use of command line driven applications. As so-called “benchtop biologists”, the use of Geneious and CLCGW was easy to learn and their cost was acceptable in view of the time saved in learning the use of command line driven programs. That said, our success was probably attributable to the small genome sizes of plant viruses, particularly BYMV and ClYVV, which are both c. 9535 nt long. Larger genomes, from unpurified RNA samples would undoubtedly be much harder to piece together, but not impossible. We found in most cases (17 out of 23) there was sufficient average coverage to be confident of good genome representation for the isolate sequenced. These sequences had average coverages as low as 65 and 457 with remaining average coverages being greater than 737 and up to 12,313 times when mapped back to a reference sequence using Geneious. Currently, sequencing a human genome of approximately 300 MB on an Illumina platform requires 30 times coverage to be adequate [31]. Therefore, it seems reasonable to designate our virus genomes with less than 30 times coverage as draft sequences. Although not meeting minimum requirements for average coverage, they are still valuable data sets, particularly given the low numbers of complete or nearly complete BYMV genomes available (now 32 including those from this study).

The settings used in *de novo* assembly are sufficient to distinguish between more than one strain or group of a plant virus when present in the same sample, as previously demonstrated by Kehoe *et al.* [9]. In our case, the sample from a *V. faba* BYMV isolate (FB) retrieved from the culture collection also contained a nearly complete LP isolate genome. The contamination probably occurred more than ten years ago when they were maintained next to each other in the same glasshouse prior to freeze-drying and storage in the collection. In such instances, if we had only been using Geneious to map to a reference genome, we would have likely missed the second sequence. It is therefore important to perform *de novo* assembly, as well as mapping to a reference genome. In cases where either the mapping or the *de novo* sequence had a gap, it was usually resolved after alignment with the sequence from the second program. However, for genomes with coverage less than ten (i.e. the draft genomes) this method was ineffective.

The uptake of NGS amongst plant virologists is increasing as the cost associated with it decreases [1]. The relatively small genome size of plant viruses allows us the opportunity to extract complete or nearly complete genomes using commercial packages. Use of NGS does raise concerns regarding the consequences of an increase in the discovery of virus or virus-like sequences. As such, MacDiarmid *et al.* [32] made recommendations regarding the identification of plant viruses through NGS, and the potential biosecurity issues associated with this. One of the recommendations was that the term “uncultured virus” should be used with any plant virus sequence not associated with a recognized virus infection. We support this recommendation whole-heartedly.

We know of no recommendation regarding requirements for depth of coverage for plant virus genomes, particularly ones involving new virus discoveries. Until such time as an appropriate set of comparative studies are done, we would recommend following in the path of our human genome colleagues by requiring a minimum coverage of at least 30 times, but this would likely lead to many nearly complete or draft plant virus genomes. However as with BYMV for example, we required coverage well into the 1000's to ensure a complete genome (including 5′ and 3′ UTRs, a constant challenge for plant virologists). Our samples sent for sequencing were total RNA, so different methods of sample preparation might have increased the numbers of virus reads. For example, use of subtractive hybridization [4], or extracting for dsRNA first, followed by random cDNA synthesis [1], [33]. Despite this, there is no doubt that NGS has been an exceedingly useful tool for our study.

## Supporting Information

Table S1 Nucleotide percentage similarities of the coding regions of thirty three *Bean yellow mosaic virus* and two *Clover yellow vein virus* isolates, calculated in MEGA 5.2.1 using a pairwise comparison with the number of differences model.
(DOCX)
