# Supplementary material for: Plant Virology and Next Generation Sequencing: Experiences with a Potyvirus
Source: PLoS One. 2014 Aug 7;9(8):e104580. doi: 10.1371/journal.pone.0104580 (PMC4125191; doi:10.1371/journal.pone.0104580)
Supplement: Table S1 — Nucleotide percentage similarities of the coding regions of thirty three Bean yellow mosaic virus and two Clover yellow vein virus isolates, calculated in MEGA 5.2.1 using a pairwise comparison with the number of differences model. (DOCX) [file pone.0104580.s001.docx]

**Supplementary Table 1.** Nucleotide percentage similarities of the coding regions of thirty three *Bean yellow mosaic virus* (BYMV) and two *Clover yellow vein virus* (ClYVV) isolates.
